# Perceived Needs of Parents Caring for Children With Cancer in Saudi Arabia: A Cross-Sectional Study

**DOI:** 10.1155/jonm/2395174

**Published:** 2025-11-06

**Authors:** Hanan Alshammari, Hebah Almulla, Fatimah A. Alnass, Sama S. Hammad

**Affiliations:** ^1^King Fahad Specialist Hospital-Dammam, Dammam 32253, Saudi Arabia; ^2^Fundamentals of Nursing Department, College of Nursing, Imam Abdulrahman Bin Faisal University, Dammam 34221, Saudi Arabia

**Keywords:** cancer, children, cross-sectional, family needs, parents, perceived needs, quantitative, Saudi Arabia

## Abstract

**Aims:**

Although global research highlights the psychological, informational, and financial strains faced by parents of children with cancer, studies from Saudi Arabia remain scarce. This study aimed to (1) describe and assess the needs of parents caring for children undergoing cancer therapy in Saudi Arabia, (2) evaluate their requirements for additional information regarding these needs, and (3) identify illness-related and demographic factors that predict the demand for this information.

**Methods:**

A total of 130 parents of children diagnosed with cancer and undergoing active treatment were recruited from a tertiary cancer hospital in Saudi Arabia for this descriptive, cross-sectional study. The Family Inventory of Needs-Pediatric II (FIN-PED II) was used to collect data through an online survey.

**Results:**

Parents deemed all 17 needs in the FIN-PED II necessary, with mean scores ranging from 2.68 to 3.62. The highest-priority needs were information about their child's condition and treatment received. A significant unmet need, reported by 60.8% of the parents, was guidance in helping their other children cope with the situation. Regarding additional information, 90.8% of parents wanted updates on their child's condition, and 86.9% sought treatment details. Time since diagnosis was the only significant predictor of the need for additional care information in the first 6 months of diagnosis.

**Conclusion:**

These results can be used to improve care and support for parents of children with cancer. This could also aid in detecting the perceived needs of this specific population, which was assessed for the first time using the FIN-PED II tool in Saudi Arabia. Understanding parents' priorities and unmet care needs is crucial for creating tailored interventions in Saudi Arabia.

**Summary**

• Nurses play a crucial role in understanding the needs of parents taking care of children with cancer.
• Oncology nurses specifically can assist parents by applying culturally sensitive strategies, especially during the first several months after diagnosis.

## 1. Introduction

Cancer is physically and psychologically draining. Despite advancements in pediatric cancer treatment, cancer remains a leading cause of death among children in developed countries [1]. In the U.S., the prevalence of pediatric cancer is reported to increase by 0.6% annually [2]. In Saudi Arabia, the incidence is approximately 99.83 cases per million people [3], accounting for 5.4% of all cancer cases. The most common pediatric cancer in Saudi Arabia is leukemia (30.8%), followed by brain tumors (16.7%), Hodgkin's lymphoma (16.3%), and bone cancer (6.8%) [4].

A child's cancer diagnosis is distressing not only for the child but also for caregivers, particularly parents, whose well-being and family life are significantly disrupted [5]. Parents often report emotions such as sorrow, compassion, fear, and helplessness as they witness their child endure both the illness and treatment side effects [3].

Although global research highlights the psychological, informational, and financial strains faced by parents, studies from Saudi Arabia remain scarce. However, evidence from the other Middle East countries provides important insights into parental experiences. Parents of children with cancer frequently report unmet psychological, physical, financial, and educational needs [6–8].

In Jordan, a study of caregivers of patients with cancer revealed significant vulnerability to high emotional distress, particularly anxiety and depression, when support systems failed to meet their needs [9]. Financial constraints were found to substantially worsen caregiver burden [6, 10]. Similarly, a study from Oman on caregivers of children with leukemia identified income limitations and inadequate medical information as major unmet needs [10]. Al-Gamal et al. [11] reported that caregiver burden extends well beyond the treatment period, with long-term psychological strain persisting for many families.

Arabiat and Altamimi [12] reported families in Jordan often struggle with insufficient communication from healthcare providers, especially regarding diagnosis and treatment. The lack of clear and timely information increases stress and disrupts family coping. Together, these studies highlight recurring themes across the Middle East: parents consistently identify gaps in emotional support, financial assistance, and access to reliable medical information, all of which directly affect their caregiving burden and quality of life.

Comparable findings have been reported from Europe, where parents expressed high demands for emotional support (55%), financial assistance (30%), and medical information (70%) [13–15]. Taken together, these studies suggest that unmet needs in pediatric oncology are a global phenomenon, though regional and cultural differences shape how they are experienced and addressed.

At the family level, unmet needs affect not only parents but also siblings. Owing to the increased attention paid to the child with cancer, siblings may face emotional difficulties. Therefore, establishing effective interventions to address the entire family's needs is crucial [16–18].

Lövgren et al. [19] revealed widespread dissatisfaction among parents and siblings regarding the quantity and quality of information available on the psychological impact of cancer treatment. Researchers have identified several factors, including family-related, sociodemographic, and illness-related factors, that predict parents' willingness to seek additional information regarding their child's diagnosis, which in turn can help them make informed decisions regarding their child's condition [20].

Despite the increasing recognition of these challenges worldwide, research on the unmet needs of Saudi parents remains limited. Currently, Saudi Arabia lacks designated supportive care centers or personnel specifically addressing parental needs [21]. Consequently, many caregivers' psychological and informational needs are overlooked, hindering long-term adaptation. This study addresses this gap by investigating the unmet needs of parents caring for children with cancer in Saudi Arabia, with particular attention to deficiencies in the existing support system.

### 1.1. Theoretical Framework: Fulfillment Theory

This study is grounded in the Fulfillment Theory, which posits that an individual's satisfaction depends on the extent to which their needs are met [22]. Unmet needs become significant psychological stressors, particularly in the context of life-threatening illnesses. For parents of children with cancer, overwhelming caregiving responsibilities often make it difficult to identify or articulate their needs, even as they face critical situations with uncertain outcomes. Therefore, nursing interventions are critical for meeting these needs by providing explanations, emotional reassurance, and practical guidance throughout the treatment process.

**Figure 1:**
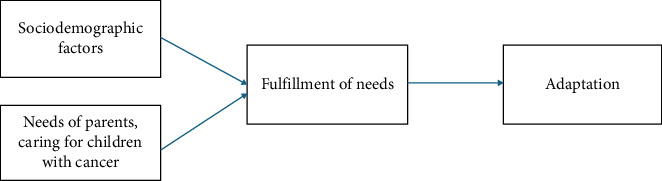
Reconceptualized framework of the Fulfillment Theory.

The reconceptualized framework of the Fulfillment Theory (Figure 1) illustrates how sociodemographic and illness-related factors, such as time since diagnosis and access to comprehensive information, interact to shape parents' needs. These factors influence whether needs are fulfilled, and in turn, affect parental adaptation and psychological well-being. This framework highlights how gaps in information and support exacerbate caregiver distress, underscoring the importance of systematic needs assessments.

Pediatric oncology, parents often report unmet needs related to understanding the diagnosis, prognosis, treatment options, and anticipated outcomes. The Family Inventory of Needs-Pediatric II (FIN-PED II) questionnaire, specifically developed based on the Fulfillment Theory [23], is designed so that the FIN-PED II acts as both a measurement instrument and a framework for interpreting the study findings.

It is a comprehensive and practical tool that captures a wide range of parental needs, encompassing emotional, informational, and support-related dimensions in a concise format.

Compared to other assessment instruments, such as the Family Inventory of Needs and the Needs of Parents Questionnaire, the FIN-PED II is distinguished by its simplicity, cultural adaptability, and robust psychometric properties. While other tools primarily focus on inpatient care or specific phases of illness, the FIN-PED II is applicable across various stages of caregiving.

This study therefore aimed to describe the needs of parents caring for children undergoing cancer in Saudi Arabia. Moreover, we aimed to evaluate parents' requirements for additional information regarding these needs and to identify illness-related and demographic factors predicting parents' demands for further information.

By addressing these objectives, this study intended to contribute critical knowledge on the unmet needs of Saudi parents and provide evidence to guide healthcare professionals and policymakers in developing targeted support systems that enhance parental and child well-being.

## 2. Materials and Methods

### 2.1. Study Design and Sampling

This quantitative cross-sectional study was conducted from December 2023 to May 2024 and adhered to the STROBE guidelines [24]. Participants were recruited using convenience sampling from King Fahad Specialist Hospital (KFSH), Dammam, Saudi Arabia, a specialized center for oncology patients. Although convenience sampling may introduce selection bias and limit representativeness, our study site, KFSH, is one of the two main oncology referral centers in the Eastern Province of Saudi Arabia. This strengthens the relevance of the findings while acknowledging that they may not be generalizable to all settings.

Eligible participants were approached in the pediatric oncology daycare, inpatient, and intensive care units. The inclusion criteria were being a parent (mother/father) of a child undergoing active cancer therapy and the ability to complete the survey in Arabic or English. Parents unable to fully understand or engage in the study were excluded.

Sample size was calculated using G∗Power. For multiple linear regression with 12 tested predictors, the alpha level of 0.05, a moderate effect size (*f*^2^) of 0.15, and a power of 0.8, the minimum sample size required was 127 participants. The moderate effect size was selected based on Cohen's conventions for multiple regression effect sizes, which are commonly applied in psychosocial and nursing research, to balance feasibility with the detection of meaningful associations [25].

### 2.2. Data Collection

Data were collected between January and March 2024. Eligible participants were identified by the principal investigator (PI) using a standardized screening checklist that included the child's confirmed cancer diagnosis, current active treatment, and parents' ability to complete the survey. To ensure uniformity, all recruitment and screening were conducted solely by the PI, who was present in the hospital daily throughout the data collection period. This minimized variability and maintained close oversight of participant enrollment. Eligible participants were identified by the PI using a standardized screening checklist that included the child's confirmed cancer diagnosis, current active treatment, and parents' willingness and ability to complete the survey.

Participants were either given the QR code of the online survey to scan and complete at their own pace or were provided an iPad to complete the online survey. The PI maintained a distance while the participants completed the survey to ensure privacy and avoid social desirability bias. We conducted a pilot study with 12 participants to assess clarity, accessibility, and administration time before launching the full-scale study. All 12 participants completed the survey within approximately 12 min. No concerns regarding item wording or technical usability were reported. Thus, no modifications were required before the main data collection.

### 2.3. Study Measures

#### 2.3.1. FIN-PED II

The FIN-PED II is a 17-item scale that measures the different care needs of parents of children with cancer [22, 26]. It comprises three subscales. The first subscale, referred to as the “Importance of Care Needs,” is rated using a five-point Likert scale with scores ranging from 0 (not at all important) to 4 (extremely important). The average score was calculated from the total number of items rated between 1 and 4. The second subscale measures the fulfillment of each identified need on a 4-point Likert scale ranging from 1 (not met at all) to 4 (completely met). The third subscale assesses the need for additional information regarding each identified need on a five-point Likert scale, with values from 0 (not at all) to 4 (a great deal). We used the Arabic version of the FIN-PED II developed and validated by Arabiat et al. [26] without revalidation, as this was beyond the scope of this study. To ensure reliability within our dataset, we calculated the Cronbach's alpha coefficient for each subscale, all of which demonstrated good internal consistency.

Monterosso et al. [22] reported adequate internal consistency reliability of 0.83, 0.90, and 0.98 for the three subscales of the FIN PED II, respectively.

Furthermore, the Arabic version also showed acceptable internal consistency reliability rate (0.70 for all three subscales) [26]. Permission to use the scale was obtained from the original authors [22, 26].

In addition, parent-related information (relationship to the child, number of other children, educational level, employment status, parents' age, marital status, and area of residence), child's demographic data (age and sex), and child's illness-related information (cancer diagnosis, time since initial diagnosis, cancer type, and type of treatment) were collected through parent self-reporting in the survey questionnaire.

### 2.4. Ethical Considerations

Prior to data collection, approval was obtained from the Institutional Review Board (IRB) of Imam Abdulrahman bin Faisal University (IRB-PGS-2024-04-007) and King Fahad Specialist Hospital, Dammam (NED0342). The data collection tool was posted online in Arabic and English as a web-based survey designed using QuestionPro. The first page of the survey included a Participant Information Sheet (PIS), which provided detailed information about the study, such as the purpose of the study, eligibility criteria, advantages and disadvanages of participating, the voluntary aspect of participation, and the option to withdraw from the study at any point without any negative consequences. Informed consent was obtained electronically. Participants were required to tick three boxes confirming that they had (1) read and understood the PIS, (2) understood that their data would be stored confidentially, and (3) voluntarily agreed to participate in the study. Only after confirming these three statements were they able to initiate the survey.

Additionally, considering that this topic had a risk of triggering memory-related trauma or causing psychological distress among participants, participants were provided with the contact information of the National Center for Mental Health Promotion if they needed any assistance or support [27]. None reported using these services, and no follow-up for psychological distress was performed, as this was beyond the scope of the study.

### 2.5. Data Analysis

The data were analyzed using IBM SPSS Statistics (Version 23). Descriptive statistics (frequencies, means, and standard deviations) were computed to report parent and child demographics and child's illness-related information. Average mean scores were calculated for all three subscales. Multiple linear regression with the enter method was used to assess significant predictors for the need for more information, which was our dependent variable. The normality, linearity, and homoscedasticity of the residuals were examined for each model to ensure that the linear regression model assumptions were tenable [28]. Furthermore, Pearson correlation analysis was computed among all predictors in the regression model to ensure the absence of multicollinearity, and a two-tailed *p* value of less than 0.05 was considered significant. Multicollinearity was assessed using the variance inflation factor (VIF), tolerance values, and collinearity diagnostics (condition index and variance proportions). VIF < 5, tolerance > 0.20, and absence of two or more predictors with variance proportions > 0.50 on the same high condition index dimension (> 30) were considered acceptable thresholds.

## 3. Results

### 3.1. Sample Characteristics

Of 153 eligible participants invited, 130 completed the survey. Nonparticipation was primarily owing to time constraints and caregiving responsibilities during medical procedures. Most respondents were fathers (*N* = 86, 66.2%), married (*N* = 124, 95.4%), and Saudi nationals (*N* = 112, 86.2%), with a mean age of 37.7 years. Half of the parents held a bachelor's degree (*N* = 65, 50.0%), while 61 (46.9%) had a high school education or less.

Mean age of the children was 7.5 years (SD = 3.9), and most were male (*N* = 87 66.9%) and diagnosed with leukemia (*N* = 84, 64.6%). Chemotherapy was the predominant treatment (*N* = 129, 99.2%). Just over half of the children (*N* = 73, 56.2%) had been diagnosed within the past year.  Table 1 provides a detailed summary of the sample characteristics.

#### 3.1.1. Important Parents' Care Needs

The three subscales of the FIN-PED II demonstrated adequate internal consistency reliability, with alpha coefficients of 0.84, 0.86, and 0.89 for the importance of needs, satisfaction of needs, and need for additional information subscales, respectively. The average score for importance of care needs was 56.6 (SD = 7.9; range, 38–68). The care needs listed in Table 2 are organized from the highest to lowest based on mean scores and standard deviations. All 17 items in the FIN-PED II were deemed necessary by the parents, with mean scores ranging from 2.68 (SD = 1.38, 95% confidence interval [CI; 2.44, 2.92]) to 3.62 (SD = 0.59, 95% CI [3.52, 3.73]). When ranked, the most critical needs were the need for information about changes in the child's condition (mean = 3.62, SD = 0.59, 95% CI [3.52, 3.73]) and the need to understand the treatment the child was receiving (mean = 3.62, SD = 0.69, 95% CI [3.50, 3.74]). The need for guidance on what information to share with siblings was rated the lowest (mean = 2.68, SD = 1.38, 95% CI [2.44, 2.92]). Detailed descriptive statistics for each item are presented in Table 2.

#### 3.1.2. The Extent of Care Needs Met by Healthcare Providers

We aimed to assess how effectively healthcare providers address the care needs of parents of children with cancer. We categorized the responses into two sections: “well met/completely met” and “partially met/not met.” The most successfully met needs, reported by 104 participants (80%), included trust in the healthcare system and confidence that healthcare professionals are genuinely committed to the child's care. Additionally, 79.2% of parents (*N* = 103) felt well informed about their child's treatment. The most commonly unmet need, reported by 79 participants (60.8%), was guidance to address their other children's feelings about the situation, followed by the need for clarity regarding what information to share with their other children (*N* = 66; 50.8%) and how to provide this information (*N* = 66; 50.8%). Other notable unmet needs included insights on when to expect side effects and what they might be, both noted by 54 participants (41.5%) (see Table 2).

#### 3.1.3. The Need for Additional Information

We assessed the 17 items related to the parents' needs to determine whether they required additional information regarding their perceived needs. Responses indicating “very much” and “a great deal” were combined into a single category. The results revealed a significant demand for additional information across all 17 needs, with at least 61.5% of the participants considering it important. The highest need for additional information was related to parents being informed about modifications to their child's condition, as indicated by 118 participants (90.8%). Following that, 113 participants (86.9%) expressed the need to know what treatment their child is receiving. In addition, 105 participants (80.8%) stated that they wanted to be informed about when and why changes are being made to their child's treatment plans.

Multiple regression analysis was used to examine whether the selected demographic and illness-related variables significantly predicted parents' perceived need for additional information. The predictors included parents' age, education level, relationship to the child, child's age, time since diagnosis, and treatment type. The collinearity diagnostics indicated no evidence of problematic multicollinearity. All VIF values were < 5 and tolerance > 0.20. The maximum condition index was 24.53, and although parent education and treatment type showed higher variance proportions (0.94 and 0.67, respectively), no two predictors exceeded 0.50 on the same dimension, confirming the stability of the regression estimates.

The overall regression model was not statistically significant (*F* (8, 121) = 1.636, *p*=0.121) and accounted for 9.8% of the variance in informational needs (*R*^2^ = 0.098; adjusted *R*^2^ = 0.038). Despite the model's non-significance, time since diagnosis of < 6 months was a statistically significant individual predictor (*β* = 0.284, *p*=0.004), indicating that parents whose children had been diagnosed within the past 6 months reported higher informational needs than those whose children had been diagnosed over a year ago. No other variables were statistically significant predictors.

## 4. Discussion

Families of children diagnosed with cancer have unique needs to cope with the burden of disease. Our study provides information on the perceived needs of parents of children with cancer, and to our knowledge, this is the first study in Saudi Arabia using the FIN-PED II [22, 26]. The aims of this study were threefold: first, to describe the most important needs of parents caring for children undergoing cancer therapy; second, to evaluate parents' requirements for additional information regarding their identified care needs; and third, to identify illness-related and demographic variables that may predict parents' demand for additional information. As this is the first such study in the Saudi Arabian population, supporting research is limited, and related studies from the region or elsewhere were used. However, some comparisons were challenging, owing to population and cultural differences.

Regarding the first subscale, all the needs in the FIN-PED II were deemed important by the parents in our study. The most important needs were that parents wanted to be informed about changes in their child's condition and to understand their treatment. This aligns with findings from similar studies conducted in Saudi Arabia and other Middle Eastern countries [7, 8, 12]. The items ranked lowest were handling the feelings of their other children and what information to give them and how. This makes sense in the Saudi Arabian culture, where family support is highly valued and is associated with a better quality of life [29]. When a child becomes ill, siblings have support from aunts, uncles, grandparents, houseworkers, and other close family members or friends. Therefore, parents need not prioritize their other children's needs. Such cultural norms align with the National Family Strategy, which envisions families grounded in solid values to make informed decisions and fulfil their potential through a helpful and cohesive family unit [21]. A study in Indonesia, which shares similar cultural values, reported that parents of children with cancer expressed that the whole family shared the sadness but eventually accepted the situation as God's will [30].

Understanding the family's needs and values has been reported as the most crucial need in Saudi Arabia [3]. Another study from Saudi Arabia revealed that providing details of the treatment, reasoning, and timing of any changes to the care plan, and prognosis were important needs [7]. Their findings align with ours, indicating that the three most crucial aspects for improving support are developing improved communication protocols, implementing tailored support programs, and encouraging healthcare professionals to undergo tailored training programs to better understand the specific needs of family caregivers [7].

The Fulfillment Theory indicates that a person's level of satisfaction depends on how well their needs are met [31]. Parents prioritize specific needs, as measured by the FIN-PED II, indicating that their involvement significantly influences the child's trajectory. Therefore, providing sufficient information related to cancer is essential not only for addressing parents' needs but also for their satisfaction and coping. In the context of caring for a child with cancer, a review suggests that providing adequate patient education is associated with reduced psychological distress throughout the illness and improved adaptation to disease progression [32].

Parents are involved in their child's treatment journey and are coping with treatment care plans and expected side effects, as well as their child's prognosis and possible recurrence of the disease [33]. Thus, healthcare providers need to consider parents' level of involvement in their child's treatment plan, as this will assist them in promoting active participation.

Regarding the second aim, the findings related to parents' requirements for additional information regarding their identified needs revealed that the most frequently met needs were trust in the healthcare system and the perception that healthcare providers were genuinely committed to their child's care. In contrast, Thomas et al. [34] reported that parents underutilized access to supporting information, emotional support, and guidance from healthcare services, and thus lacked trust in the system. However, the situation may be different in Saudi Arabia. Since the launch of National Vision 2030, tremendous advancements, especially through the Quality of Life Program, have led to an increase in the general public's confidence and trust in the healthcare system [35].

Parents' confidence in the healthcare system is critical in patient care. Likewise, understanding parents' needs is important for healthcare providers to provide appropriate support, thereby facilitating a smooth caregiving experience at home. The compassion exhibited by healthcare providers holds significant importance in the perception of patients and parents regarding the quality of pediatric care [36]. A study from Oman found that unmet needs increased over time, such as discussions with healthcare professionals and obtaining information. Therefore, the healthcare team should continuously provide caregivers comprehensive information as the illness advances, including anticipated signs and immediate and lasting consequences of cancer therapy [10].

In our study, the most unmet need was guidance on addressing the feelings of siblings. Although this was the lowest-ranked need in our study, parents still expressed it as an unmet need. Similar to our study, a review showed that support for siblings was among the most unmet needs [37]. This area can be a focus for future research, particularly in the treatment plan of families in Saudi Arabia. Families in Saudi Arabia tend to have houseworkers who become part of the family unit, and comparative research is lacking on the various experiences of families [38]. Healthcare providers should ask parents about their other children and whether they require support in this regard, referring them to appropriate counseling. When examining the extent of care needs met by healthcare providers in our study, parents expressed a need for instructions on providing information to other children, when to expect side effects, and what the side effects were. Given that these items were considered important in our sample, they may serve as focus areas for future projects and hospital interventions to support parents and caregivers in Saudi Arabia. Moreover, variations in parental experience and nursing requirements across different treatment phases for children with cancer highlight the need for phase-specific educational interventions. Such tailored programs can enhance practical support for parents of pediatric cancer patients, as they align with the Fulfillment Theory to better guide these interventions.

Regarding the third aim, although the overall regression model was not significant, “time since diagnosis within the first 6 months” was a significant predictor. During this period, families may face emotional and informational challenges when adjusting to the new health conditions and making complex treatment decisions for their child. The lack of significant associations with other demographic or illness-related variables indicates that information needs are influenced less by these fixed factors and more by stress at the beginning of the disease process. Practitioners should understand that parents require additional attention during the first months of diagnosis. One study emphasized that parents greatly need information about their child's cancer during this period, in addition to psychological and emotional support [15].

Overall, our study revealed a significant demand for additional information across all 17 items in PED-FIN II, especially regarding treatment plans and changes. These findings highlight the need for culturally sensitive care plans tailored to individual family needs and may help future hospital interventions. Building trust and offering parents genuine support during this period requires improved communication skills and listening to families with empathy [6].

Nursing interventions grounded in the Fulfillment Theory effectively reduce the impact of childhood cancer on families and facilitate coping [39, 40]. The need for additional information implies that families perceive a gap in their knowledge, warranting further research on the relationship between families' interactions with the healthcare system and their child's outcomes. As families' specific needs differ based on cultural norms and emotional and situational contexts, theoretical models such as the Fulfillment Theory can help design tailored nursing interventions, not only to address the gaps in knowledge but also to support parents to engage in a meaningful journey in their child's treatment.

### 4.1. Strengths and Limitations

This study, the first in Saudi Arabia to use the FIN-PED II, explored the unique needs of parents caring for children with cancer. The instrument showed strong internal consistency. However, it is important to acknowledge the following limitations. First, this descriptive cross-sectional study was conducted at a single hospital in the Eastern Province of Saudi Arabia, which may limit the generalizability of the findings. Nonetheless, the selected investigation site is the sole pediatric tertiary referral hospital in the region, treating the majority of pediatric oncology cases and providing the sole radiation therapy service in the area. Thus, parents' perceived needs may vary across institutions based on the range of services offered. Second, the nature of cross-sectional studies introduces an increased risk of potential bias from self-reported data, such as social desirability or recall bias, which could affect the accuracy and objectivity of the participants' responses. Third, owing to the nature of the design, the collection of longitudinal follow-up data to assess the changes needed over time was lacking. Parental needs can evolve, especially since nearly half of the participants had long-term follow-up exceeding 1 year, while the rest had follow-ups for less than a year. Fourth, convenience sampling based on participant availability may have introduced a selection bias and limits generalizability. Finally, the gender imbalance in the sample can be explained by Saudi Arabian cultural norms, where fathers are generally responsible for accompanying their children, especially those with chronic illnesses, to hospital appointments. This may have influenced gender distribution and potentially impacted the findings.

### 4.2. Implications for Policy and Practice

The results of our study have important implications for clinical practice and policies. Parents of children with cancer require sufficient information about treatment details and updates on their child's condition, especially during the first 6 months. Meeting parents' and caregivers' needs during the treatment journey should be prioritized. Future policy changes in oncology care should include support services for parents to facilitate coping and mitigate psychological distress [10]. Targeted training programs on psychosocial care for siblings should be considered, with emphasis on culturally tailored interventions involving the extended family, rather than just the affected child, and spirituality [30]. Further implications for practice include using electronic health records to integrate screening measures, such as assessment of family burden and identification of at-risk parents. Screening is crucial both at admission and at various time points and follow-ups [6]. These efforts can help improve the quality of life of families and alleviate the burden on parents at high risk; therefore, healthcare institutions are encouraged to offer interventions tailored to meet families' unique needs.

Although families felt the sincerity of healthcare professionals in this study, the identified unmet needs should be considered when developing tailored interventions for families in Saudi Arabia. Moreover, future longitudinal studies with follow-ups should be undertaken for a deeper understanding of this topic. Strategies should be developed to achieve a more balanced representation of both mothers and fathers in studies, to account for differences between parents. In Saudi Arabia, oncology treatment is primarily provided in government specialty hospitals, free of charge; however, future studies should incorporate socioeconomically diverse samples from both government and private hospitals to investigate whether perceived needs differ across socioeconomic levels.

## 5. Conclusion

Parents in Saudi Arabia expressed a need for clear guidance in managing the emotional needs of siblings, including providing them age-appropriate information about the illness and treatment. Therefore, tailored support programs should be developed based on the unique needs of each family, to address the emotional and developmental needs of siblings, in line with the guiding theory. Implementation of such programs will enable nurses and other healthcare professionals to support families and parents in caring for children with cancer. Oncology nurses are at the forefront of supporting parents' wellbeing and can implement culturally sensitive interventions once the specific needs of families and parents are known.

Culturally sensitive educational materials for families can aid in recognizing and responding to stress in the family. Our findings support the Fulfillment Theory by illustrating how unmet needs regarding siblings, such as emotional support, recognition, and autonomy, represent gaps in psychological fulfillment. Moreover, this study extends the theory's application to pediatric oncology within the Saudi Arabian cultural context, emphasizing the need for culturally tailored interventions that promote holistic family well-being.

In summary, actionable recommendations from this study include, first, creating culturally sensitive educational materials that provide siblings with age-appropriate information about the illness and treatment. Second, structured communication protocols should be established to inform families about the side effects of treatment and their timing. Third, family specific support programs that address their emotional, informational, and practical needs, based on individualized family assessments, should be implemented. Fourth, oncology nurses should be trained in a culturally sensitive manner to ensure that interventions align with Saudi Arabian family values and dynamics. Finally, psychosocial screening tools should be integrated into routine care for early identification and management of unmet needs.

## Figures and Tables

**Table 1:** Sample characteristics (*N* = 130).

Demographic information	Frequency	Percentage (%)
Child gender		
Male	87	66.9
Female	43	33.1
Type of cancer		
Leukemia	84	64.6
Lymphoma	23	17.7
Brain cancer	9	6.9
Other (sarcoma, optic nerve glioma, wilms tumor, retinoblastoma, cervical tumor, low-grade glioma)	5	3.8
Time since diagnosis		
< 1 year	73	56.2
> 1 year	57	43.8
Type of treatment		
Chemotherapy	129	99.2
Radiotherapy	14	10.8
Surgery	19	14.6
Other (nivolumab and dinutuximab immunotherapy)	2	1.6
Relationship to the child		
Father	86	66.2
Mother	44	33.8
Parent's nationality		
Saudi	112	86.2
Non-Saudi	18	13.8
Parent's marital status		
Married	124	95.4
Divorced	6	4.6
Number of children in the family		
1–4 children	95	73
5–8 children	30	23
9–12 children	5	3.9
Parent's educational level		
High school or less	61	46.9
Diploma	4	3.1
Bachelor's degree	65	50.0
Parent's employment status		
Employed	51	39.2
Unemployed	79	60.8

**Table 2:** Descriptive statistics of the care needs according to their importance.

Needs ranked		Importance of needs		Fulfillment of needs		Need for additional information		Needs not/partially met	Well/completely met	Need for information very much/great deal
		Mean	(SD)	Mean	(SD)	Mean	(SD)	*N* (%)	*N* (%)	*N* (%)
1	To be informed of changes to my child's condition	3.62	0.588	3.06	0.842	3.40	0.894	30 (23.1)	100 (76.9)	118 (90.8)
2	To know what treatment my child is receiving	3.62	0.685	3.18	0.887	3.21	1.212	25 (19.2)	103 (79.2)	113 (86.9)
3	To feel there is hope	3.55	0.705	2.88	0.845	2.89	1.247	41 (31.5)	89 (68.5)	99 (76.2)
4	To feel that the healthcare professionals are sincere in caring about my child	3.52	0.662	3.11	0.900	2.85	1.344	26 (20.0)	104 (80.0)	97 (74.6)
5	To have trust in the healthcare system	3.49	0.696	3.08	0.778	2.79	1.310	26 (20.0)	104 (80.0)	92 (70.8)
6	To know the probable outcome of my child's illness	3.49	0.707	2.94	0.842	3.02	1.217	32 (24.6)	97 (74.6)	102 (78.5)
7	To have thorough information about how to care for my child at home	3.47	0.779	3.02	0.889	2.94	1.322	32 (24.6)	97 (74.6)	94 (72.3)
8	To know what side effects the treatment can cause.	3.47	0.728	2.65	0.834	2.93	1.234	54 (41.5)	75 (57.7)	92 (70.8)
9	To be told when and why changes are being made in my child's treatment plans	3.41	0.775	3.04	0.848	3.01	1.217	32 (24.6)	98 (75.4)	105 (80.8)
10	To know when to expect side effects to occur	3.40	0.784	2.58	0.896	2.96	1.284	54 (41.5)	75 (57.7)	90 (69.2)
11	To have explanations given in terms that are understandable to me	3.40	0.722	3.11	0.891	2.92	1.245	27 (20.8)	102 (78.5)	96 (73.8)
12	To know I can ask questions any time	3.38	0.719	3.00	0.863	2.75	1.330	34 (26.2)	95 (73.1)	95 (73.1)
13	To know to whom I should direct my questions	3.23	0.859	2.82	0.927	2.83	1.313	39 (30.0)	89 (68.5)	97 (74.6)
14	To know that healthcare professionals will offer me the opportunity to participate equally in my child's care	3.12	0.886	2.92	0.881	2.75	1.259	38 (29.2)	90 (69.2)	91 (70.0)
15	To know how to handle the feelings of my other children	2.89	1.31	1.79	1.112	2.78	1.501	79 (60.8)	37 (28.5)	89 (68.5)
16	To know how to give information to my other children (appropriate to his/her age)	2.87	1.37	1.96	1.26	2.57	1.530	63 (48.5)	50 (38.5)	82 (63.1)
17	To know what information to give to my other children (appropriate to his/her age)	2.68	1.37	1.88	1.21	2.48	1.531	66 (50.8)	45 (34.6)	80 (61.5)

## Data Availability

The data that support the findings of this study are available from the corresponding author upon reasonable request.
